# MRI-visible enlarged perivascular spaces in basal ganglia rather than centrum semiovale was associated with aneurysmal subarachnoid hemorrhage

**DOI:** 10.3389/fneur.2024.1341499

**Published:** 2024-01-15

**Authors:** Qiuyue Yu, Haichao Wang, Wenyi Zhang, Xiang Zhang, Jingjing Zhao, Li Gong, Xueyuan Liu

**Affiliations:** ^1^Department of Neurology, Tongren Hospital, Shanghai Jiao Tong University School of Medicine, Shanghai, China; ^2^Department of Neurology, Shanghai Tenth People's Hospital, Tongji University, Shanghai, China; ^3^Department of Anesthesiology, Tongren Hospital, Shanghai Jiao Tong University School of Medicine, Shanghai, China; ^4^Department of Neurosurgery, Shanghai Tenth People's Hospital, Tongji University, Shanghai, China

**Keywords:** subarachnoid hemorrhage, aneurysm, stroke, enlarged perivascular spaces, glymphatic system

## Abstract

**Background:**

The subarachnoid space is continuous with the perivascular compartment in the central nervous system. However, whether the topography and severity of enlarged perivascular spaces (EPVS) correlates with spontaneous subarachnoid hemorrhage (SAH) remains unknown. Based on the underlying arteriopathy distributions, we hypothesized that EPVS in basal ganglia (BG-EPVS) are more closely associated with aneurysmal subarachnoid hemorrhage (aSAH) than other SAH without aneurysm.

**Methods:**

Magnetic resonance imaging (MRI) scans of 271 consecutive SAH survivors with and without aneurysm were analyzed for EPVS and other markers of imaging data. In the subgroup analysis, we compared the clinical characteristics and EPVS of SAH participants with and without pre-existing known risk factors (hypertension, diabetes, and smoking history) using multivariable logistic regression.

**Results:**

Patients with aSAH (*n* = 195) had a higher severity of BG-EPVS and centrum semiovale EPVS (CSO-EPVS) than those without aneurysm (*n* = 76). Importantly, BG-EPVS predominance pattern (BG-EPVS>CSO-EPVS) only existed in aSAH survivors rather than other SAH without aneurysm. In the subgroup analysis, interestingly, we also found that a high degree of BG-EPVS showed an independent relationship with aSAH in patients without pre-existing risk factors (e.g., hypertension).

**Conclusion:**

In this cohort study, BG-EPVS predominance pattern was associated with aSAH patients compared with those without aneurysm. Moreover, BG-EPVS still showed a strong association with aSAH survivors without pre-existing vascular risk factors. Our present study suggested the BG-EPVS as a potential MRI-visible characteristic would shed light on the pathogenesis of glymphatic function at the skull base for aSAH.

## 1 Introduction

Subarachnoid hemorrhage (SAH) is a devastating disease with a high fatality rate which also causes substantial disability among survivors. Aneurysm rupture is the leading cause of spontaneous SAH, defined as aneurysmal subarachnoid hemorrhage (aSAH) (1). On the other hand, the causes of spontaneous SAH rather than aneurysm include arteriovenous malformation (AVM), anticoagulation use, vasculitis, etc. (2). The subarachnoid space is continuous with the perivascular spaces, which are interstitial fluid-filled cavities surrounding the small penetrating vessels (3). Recently, magnetic resonance imaging (MRI)-visible enlarged perivascular spaces (EPVS) emerged as a potential neuroimaging marker indicating the different underlying arteriopathy: EPVS in the basal ganglia (BG-EPVS) is associated with hypertensive arteriopathy, while EPVS in the centrum semiovale (CSO-EPVS) correlates with age-related cerebral amyloid angiopathy (CAA) (4). However, few studies have focused on the prevalence and distribution of MRI-visible EPVS in the SAH population. Therefore, we first sought to examine and compare the severity and topography of EPVS in the SAH with and without aneurysm in 2 specialist stroke centers. Moreover, as the subclinical imaging marker of cerebral small vessel diseases, the prevalence and location of EPVS would be associated with vascular risk factors. Hence, the second goal of this study was to explore whether EPVS correlated with these risk variables in SAH survivors.

## 2 Materials and methods

### 2.1 Study population

**Figure 1:**
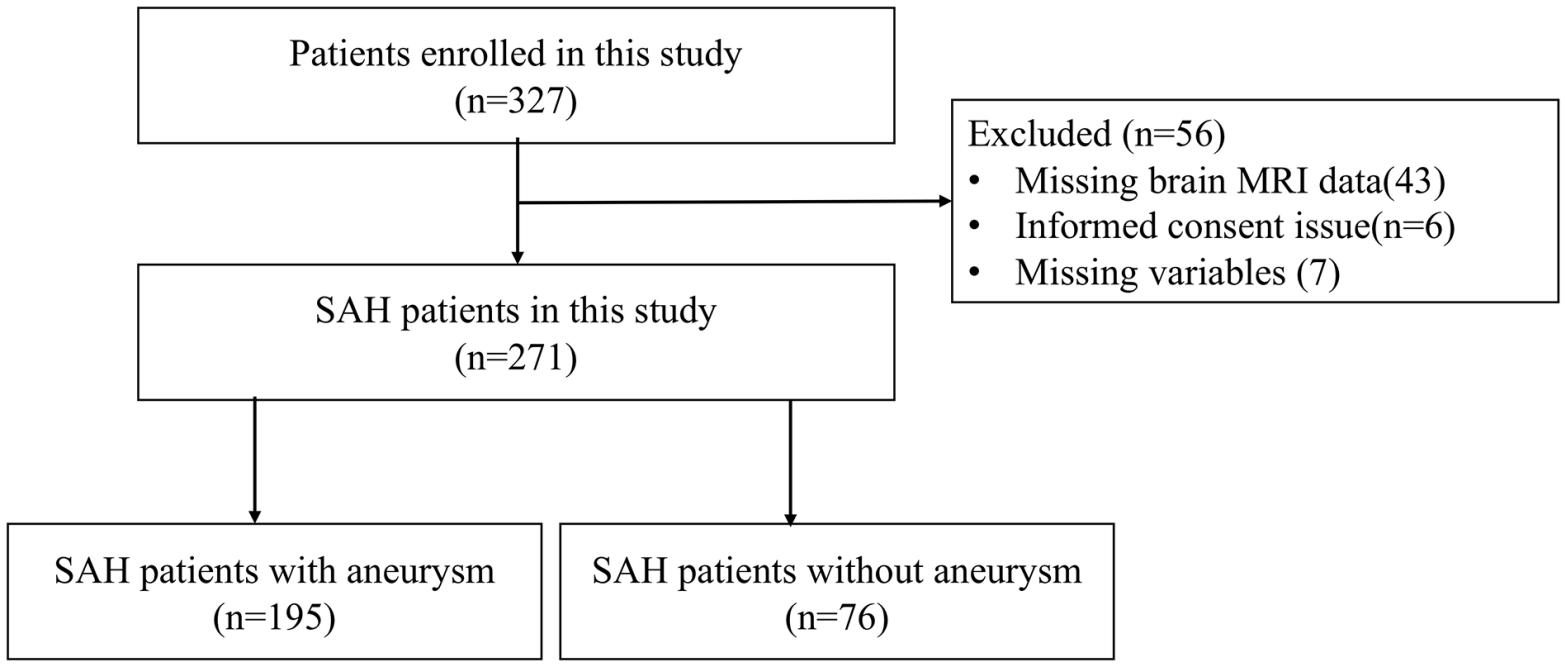
Flowchart of participant enrollment. SAH, subarachnoid hemorrhage; n, number of patients.

We analyzed data from a prospective cohort study of consecutive spontaneous SAH survivors admitted Shanghai Tenth People’s Hospital and Tongren Hospital from September 2014 to June 2020. Patients were enrolled in this study if they had spontaneous SAH and underwent MRI scans from 2 weeks to 1 month after the onset of SAH. The exclusion criteria were as follows: (1) secondary causes of SAH, such as trauma or hemorrhagic transformation of ischemic infarction; (2) unknown time of SAH onset; (3) contraindication to MRI, such as non-MRI compatible implants; (4) patients whose status is very severe or deteriorated in way they cannot perform MRI. Figure 1 shows the flow chart of participant enrollment.

We retrieved baseline clinical and demographic information, including age, sex, educational level, family history of intracranial aneurysm, spontaneous SAH onset to MRI scan time, previous history (hypertension, diabetes) and the location (anterior or posterior circulation) and size of aneurysm.

Hunt-Hess and World Federation of Neurological Surgeons SAH grading scales were used to access the severity of admission neurologic grade (5). We used the Hunt-Hess scale as the primary instrument for grading neurologic impairment, with grades 1, 2, and 3 classified as good grade and grades 4 and 5 (stupor and coma, respectively) as poor grade. The amount of blood on the computed tomography (CT) scan was classified according to the Fisher grade.

The Glasgow Coma Scale (GCS) scale, Hunt-Hess Scale, World Federation of Neurological Societies Scale (WFNS), The Modified Rankin Scale (mRS) were assessed by trained neurologists, The education level is specifically divided into illiteracy, primary school, middle school, high school, college and above, corresponding to 0, 6, 9, 12, 16 years of education, respectively.

### 2.2 Neuroimaging acquisition and analysis

All patients underwent MRI according to a standardized protocol as part of their routine clinical assessment. The protocol included T1- and T2-weighted fluid-attenuated inversion recovery (FLAIR), diffusion-weighted imaging (DWI) with 2 *b* values (0 and 1,000), and apparent diffusion coefficient (ADC) sequences. All studies were performed using 3.0-T scanners. Sequences typically included 24–30 slices of 5 mm thickness with a matrix size of 128 × 128. Digital subtraction angiography (DSA) was the only qualified measurement for determining the presence or absence of an aneurysm in this study. Diagnosis of aSAH with DSA was evaluated by two senior neurosurgeons.

As reported in previous studies (6, 7), EPVS on axial T2-weighted MRI were separated in basal ganglia and centrum semiovale regions and stratified into 3 groups: <10, 10–20, and > 20. The numbers referred to the EPVS on one side of the brain. The side or slice with the highest number of EPVS after all relevant slices for each anatomic area were reviewed. In the presence of confluent white matter hyperintensities (WMH), an estimation was made for the closest EPVS rating category. In cases of large lobar or deep SAH, EPVS was assessed in the contralateral hemisphere, the closest category ipsilateral to the lesion was estimated, and the highest severity was recorded. For this analysis, we defined high BG-EPVS or CSO-EPVS as ≥10. We also defined a composite variable containing three categories by comparing the degree of CSO-EPVS and BG-EPVS burden: a high degree of CSO-EPVS (i.e., CSO-EPVS > BG-EPVS), an equal degree in the two regions (i.e., CSO-EPVS = BG-EPVS), and a high degree of BG-EPVS (i.e., BG-EPVS > CSO-EPVS). The severity of WMH was rated according to the Fazekas scale, as previously described (8). The total Fazekas score was calculated by adding the periventricular and deep white matter lesion scores. All MRI analysis were reviewed blinded to clinical data by two trained image analysts blinded to patients’ clinical characteristics according to the STandards for ReportIng Vascular changes on nEuroimaging (STRIVE). In case of disagreement, consensus was made. (9).

### 2.3 Statistical analysis

We divided the patients with SAH into two groups according to the presence or absence of aneurysm and compared the clinical and imaging characteristics using univariate binary logistic regression. To determine whether a specific subgroup was associated with the incidence of aSAH, particularly in female survivors, we performed multivariate regression analysis. Patients were divided based on pre-existing vascular risk factors, including hypertension, diabetes, and smoking history. A *p*-value ≤0.05 was defined as statistically significant. Statistical analyses were performed using SPSS 24.0 (IBM Corp., Armonk, NY, United States).

## 3 Results

### 3.1 Baseline demographic, clinical, and neuroimaging characteristics

Among the 327 spontaneous SAH survivors, 271 patients who underwent head CT and MRI were included in the cohort (Figure 1). The main reason for exclusion (*n* = 56 patients) was that SAH survivors were too unwell to undergo MRI measurement (*n* = 43 patients), informed consent issue (*n* = 6), missing key variables (*n* = 7), Patients with spontaneous SAH who were excluded from the study were not significantly different from those included in mean age (61.42 vs. 58.53; *p* = 0.082) and female sex (50.0% vs. 60.6%; *p* = 0.115). The median time from the SAH onset to MRI was 17.5 days. Patients who were excluded had a higher Hunt-Hess grade (82.8% vs. 13.4%; *p* < 0.01).

Finally, 271 patients with SAH were included in this study. Of these, 195 (72%) patients had an aneurysm [mean age, 59.5; 122 (63%) female], and 76 did not [mean age, 54.2; 31 (41%) female]. Supplementary Table S1 summarize the clinical and radiological data of the SAH cohort obtained using univariate analysis. In comparison to SAH without aneurysm, aSAH survivors were more likely to be older, female, and have hypertension, a higher bleeding amount (Fisher grade), more severe WMH, high CSO-EPVS, and high BG-EPVS (Table 1). Overall, patients with an aneurysm were more likely to have a high degree of EPVS (≥10 EPVS) in the basal ganglia (78.5% vs. 27.6%) and in the centrum semiovale (62% vs. 34.2%) compared with other patients without an aneurysm (Table 1).

**Table 1:** Baseline demographic, clinical and neuroimaging characteristics of SAH patients with vs. without aneurysm.

Variables	With Aneurysm (*n* = 195)	Without aneurysm (*n* = 76)	*p-*value
Age, y, mean (SD)	59.5(12.8)	54.2(10.8)	**0.002**
Sex, female, n (%)	122(62.6)	31(40.8)	**0.001**
Smoking history, n (%)	69(35.4)	27(35.5)	0.983
Hypertension, n (%)	113(57.9)	29(38.2)	**0.004**
Diabetes, n (%)	46(23.6)	13(17.1)	0.247
FIA, n (%)	8(4.1)	2(2.6)	0.827
Fisher grade, n (%)			**<0.001**
Low-moderate (1–2)	63(32.3)	44(57.9)	
High (3–4)	132(67.7)	32(42.1)	
Total Fazekas score, median (range)	2(0–6)	1(0–6)	**<0.001**
CSO EPVS, n (%)			**<0.001**
<10	74(37.9)	50(65.8)	
10 ~ 20	94(48.2)	20(26.3)	
>20	27(13.8)	6(7.9)	
BG EPVS, n (%)			**<0.001**
<10	42(21.5)	55(72.4)	
10 ~ 20	84(43.1)	21(27.6)	
>20	69(35.4)	0(0)	

BG, basal ganglia; CSO, centrum semiovale; EPVS, enlarged perivascular spaces; FIA, familial intracranial aneurysm. *p* value ≤0.05, significantly.

### 3.2 EPVS predominance patterns in SAH with aneurysm and without aneurysm

**Figure 2:**
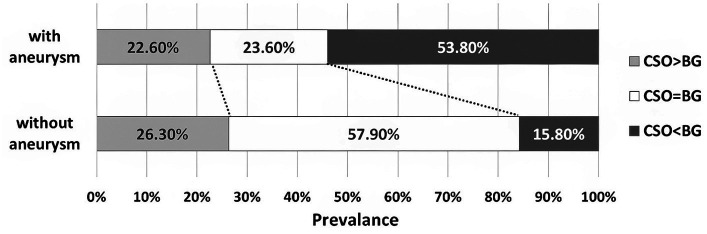
EPVS predominance patterns in SAH with aneurysm vs. without aneurysm.

The BG-EPVS-predominant pattern (BG-EPVS > CSO-EPVS) was more commonly seen in the aSAH group (53.8%) than in the other SAH patients (15.8%). The same ratio of BG-EPVS and CSO-EPVS (BG-EPVS = CSO-EPVS) was found in SAH patients with and without aneurysm (23.6% vs. 57.9%; Figure 2).

### 3.3 Relationship between EPVS and aSAH in subgroup analysis

According to pre-existing vascular risk factors, including hypertension, diabetes, and smoking history, we performed a multivariate regression analysis to determine if BG-EPVS related to a specific subgroup of aSAH survivors. As described in the subgroup analysis, BG-EPVS (OR 20.15; 95% CI 6.86–59.23; *p* < 0.001) and female gender (OR 4.26; 95% CI 1.40–12.95; *p* = 0.011) were independent factors related to aSAH survivors without hypertension (Table 2), and BG-EPVS (OR 9.92; 95% CI 4.35–22.60; *p* < 0.001), female gender (OR 4.01; 95% CI 1.79–9.00; *p* = 0.001), and hypertension (OR 3.04; 95% CI 1.32–6.99; *p* = 0.009) were independent factors related to aSAH survivors without diabetes (Table 3); and BG-EPVS (OR 33.58; 95% CI 9.89–114.07; *p* < 0.001), and female gender (OR 4.17; 95% CI 1.17–14.86; *p* = 0.028), were independent factors related to aSAH survivors without smoking history (Table 4). In the groups with pre-existing risk factors, we did not find the co-existence of female sex and BG-EPVS to be significantly associated with aSAH in multivariate analyses (Supplementary Table S1).

**Table 2:** Multivariate analysis showing variables independently associated aSAH patients without hypertension.

Patients without hypertension (*n* = 129)	OR (95% Cl)	*p*-value
Age, y	1.03(0.98–1.08)	0.221
Sex, female	4.26(1.40–12.95)	**0.011**
Diabetes	–	–
Smoking history	–	–
High Fisher grade (3–4)	1.55(0.50–4.74)	0.446
Total Fazekas score	0.76(0.48–1.22)	0.259
High CSO-EPVS (n ≥ 10)	2.78(0.80–9.69)	0.109
High BG-EPVS (n ≥ 10)	20.15(6.86–59.23)	**<0.001**

aSAH, aneurysmal subarachnoid hemorrhage; BG, basal ganglia; Cl, confidence interval; CSO, centrum semiovale; EPVS, enlarged perivascular spaces; OR, odds ratio. *p* value ≤0.05, significantly.

**Table 3:** Multivariate analysis showing variables independently associated aSAH patients without diabetes.

Patients without diabetes (*n* = 212)	OR (95% Cl)	*p*-value
Age, y	1.02(0.98–1.05)	0.386
Sex, female	4.01(1.79–9.00)	**0.001**
Hypertension	3.04(1.32–6.99)	**0.009**
Smoking history	–	–
High Fisher grade (3–4)	2.20(0.99–4.87)	0.052
Total Fazekas score	0.86(0.63–1.16)	0.324
High CSO-EPVS (n ≥ 10)	1.42(0.59–3.41)	0.433
High BG-EPVS (n ≥ 10)	9.92(4.35–22.60)	**<0.001**

aSAH, aneurysmal subarachnoid hemorrhage; BG, basal ganglia; Cl, confidence interval; CSO, centrum semiovale; EPVS, enlarged perivascular spaces; OR, odds ratio. *p* value ≤0.05, significantly.

**Table 4:** Multivariate analysis showing variables independently associated aSAH patients without smoking history.

Patients without smoking history (*n* = 175)	OR (95% Cl)	*p*-value
Age, y	1.04(0.99–1.10)	0.092
Sex, female	4.17(1.17–14.86)	**0.028**
Hypertension	2.43(0.83–7.14)	0.105
Diabetes	–	–
High Fisher grade (3–4)	1.15(0.40–3.30)	0.802
Total Fazekas score	0.88(0.58–1.33)	0.530
High CSO-EPVS (n ≥ 10)	3.11(0.98–9.82)	0.053
High BG-EPVS (n ≥ 10)	33.58(9.89–114.07)	**<0.001**

aSAH, aneurysmal subarachnoid hemorrhage; BG, basal ganglia; Cl, confidence interval; CSO, centrum semiovale; EPVS, enlarged perivascular spaces; OR, odds ratio. *p* value ≤0.05, significantly.

## 4 Discussion

In this cohort study, we found that the severity and distribution of EPVS differed according to the cause of SAH. Not only the high severity of EPVS was more common in SAH survivors with aneurysms than those without aneurysms, but also a BG-EPVS predominant pattern showed a strong independent association with aSAH survivors. Interestingly, the BG-EPVS predominant feature still exists in aSAH without pre-existing vascular risk factors. Our findings provide new evidence that the topographic pattern of BG-EPVS might be a characteristic of the underlying arteriopathy and glymphatic dysfunction in aSAH.

We found more than 70 and 60% of the high degree of EPVS (≥10) in the basal ganglia and centrum semiovale in aSAH survivors in our cohort, respectively, which is consistent with two recent retrospective studies (5, 10). In the current concept, PVS is identified as the space that surround small arterioles and venules in the brain, which has been proved to participate in process of fluid exchange and clearing waste products from the central nervous system (11). The pathological characteristic of SAH is the accumulation of extravasated blood in the subarachnoid space. As the subarachnoid space is continuous with the paravascular compartment in the brain, the event of SAH may result in the occlusion of PVS with clots formation and the disturbance of fluid drainage, finally leading to PVS extended (12, 13). This observation was evidenced in several animal studies showing that EPVS was associated with the reduced brain clearance of waste products and glymphatic dysfunction after a SAH attack occurred (14, 15).

One important finding of our study was the positive association of the BG-EPVS predominant pattern with aSAH, when we use a method of assessing EPVS predominance pattern, either CSO-EPVS, BG-EPVS or CSO-EPVS=BG-EPVS, as described by Charidimou et al. (16). In the present study, the BG-EPVS predominant pattern was found in 53.8% of the survivors with aneurysm, but it was detected in 15.8% of other SAH patients without aneurysm. A recent breakthrough in the CNS was the discovery of glymphatic system, which was identified as a route moving CSF into the brain along perivascular spaces, and eventually removing metabolic waste in the CSF to the periphery (17–19) Therefore, our present finding might be explained by a remarkable study demonstrating that basal rather than dorsal meningeal lymphatic vessels are the main route for macromolecule drainage of CSF into the peripheral lymphatic (20). We proposed that the BG-EPVS predominant pattern might reflect basal meningeal lymphatic vessels as a major exit pathway for extravasated after aSAH. Moreover, according to previous literatures, using the same EPVS rating scale as in our study, a high degree of BG-EPVS was associated with hypertensive arteriopathy, but a high degree of CSO-PVS correlated with cerebral amyloid angiopathy (16, 21). Therefore, our present study indicated the opinion that aSAH was more closely related to hypertensive arteriopathy, while other SAH without aneurysm may linked to non-hypertensive arteriopathy such as cerebral amyloid angiopathy. Notably, due to the absence of a non-SAH control group, a further study is required to directly compare the EPVS predominant pattern, or mean total EPVS score, with that of healthy controls.

Compared with intracerebral hemorrhage, CAA, and ischemic stroke, aSAH is supposed to be an earlier-onset subtype of spontaneous stroke, suggesting a higher ratio of younger patients and the absence of vascular risk factors among these patients (22, 23). Age was found to be associated with aSAH in univariate analysis, but without significance in logistic regression model. Previous study indicated that EPVS is a common occurrence in the aging population (24), but only advanced age seems to be the major risk factor associated with EPVS (25). As the age of the participants in our study mostly ranged from young to middle age (<60 years), we suppose the influence of age on EPVS was mild. To the best of our knowledge, no previous study has explored the relationship between EPVS and aSAH in patients without pre-existing risk factors. As the imaging marker of cerebral small vessel diseases, the prevalence and location of EPVS would be associated with vascular risk factors and aging. Thus, we investigated the association of EPVS with these “low-risk” aSAH survivors to minimize their effects on EPVS.

It has been demonstrated that venous insufficiency could increase perivenular edema, subsequently leading to impairment of glymphatic clearance and retention of fluid in the PVS which facilitates the onset of EPVS (26–28). Due to the fact that basal ganglia is drained by deep medullary veins only, while the centrum semiovale can be drained alternatively by cortical veins and superficial medullary veins, Min L and colleagues proposed the hypothesis of BG-EPVS but not CSO-EPVS predominant pattern that glymphatic system in the centrum semiovale could still maintain intact function under the circumstance of deep medullary veins impairment, making the PVS in the centrum semiovale higher compensatory capacity (29). Therefore, as our opinion, it might be the potential cause of BG-EPVS predominant pattern in these “low-risk” aSAH survivors that PVS in basal ganglia was more vulnerable due to decreased compensatory capacity for drainage of fluid and metabolic waste after an attack of aSAH, which encouraged the occurrence of BG-EPVS.

Strengths of our study include the standardized evaluation of MRI scans for a range of small vessel disease imaging markers, certainty of the existence of aneurysm by experienced neurosurgeons using DSA, the use of EPVS predominant patterns, and the enrollment of survivor from more than one stroke center. Our study has several limitations. First, due to the cross-sectional nature of the prospectively collected data from this cohort, it was difficult to determine the change in the occurrence of EPVS before and after SAH, as well as the causal link between them. We also acknowledge that most of excluded patients did not undergo MRI imaging due to their severe hemorrhage, which might lead to potential selection bias toward mild to moderate aSAH cases and relatively less prevalence of high degree EPVS in our study. Third, the patients without aneurysm did not undergo repeated DSA, which is needed in cases where no aneurysm was found. Although the family history of intracranial aneurysm was captured in this study, it might be another limitation that the ratio of positive history was too low to be significant in both groups. As the genetic sample was not routinely collected, future studies should consider the possibility that EPVS is genetic and might modulate the family history of aneurysm.

## 5 Conclusion

In conclusion, our study demonstrated that the BG-EPVS predominant pattern is a topographic characteristic in aSAH, as well as a positive association of BG-EPVS with aSAH survivors seemly “low risk.” This finding provided the topographic pattern of BG-EPVS as a potential MRI-visible feature, shedding light on the pathogenesis of glymphatic function at the skull base for aSAH.

## Data availability statement

The original contributions presented in the study are included in the article/Supplementary material, further inquiries can be directed to the corresponding authors.

## Ethics statement

The studies involving humans were approved by the Ethics Committee of Shanghai Tenth People’s Hospital 157 (Shanghai, China). The studies were conducted in accordance with the local legislation and institutional requirements. The participants provided their written informed consent to participate in this study.

## Author contributions

QY: Writing – original draft. HW: Data curation, Formal analysis, Writing – review & editing. WZ: Data curation, Writing – review & editing. XZ: Data curation, Writing – review & editing. JZ: Methodology, Writing – review & editing. LG: Validation, Visualization, Writing – review & editing. XL: Conceptualization, Supervision, Writing – review & editing.
