# Supplementary material for: MRI-visible enlarged perivascular spaces in basal ganglia rather than centrum semiovale was associated with aneurysmal subarachnoid hemorrhage
Source: Front Neurol. 2024 Jan 15;15:1341499. doi: 10.3389/fneur.2024.1341499 (PMC10825963; doi:10.3389/fneur.2024.1341499)
Supplement: Supplementary file 1 [file Table_1.DOCX]

| **Supplementary Table S1 Multivariate analysis showing variables independently associated aSAH patients with pre-existing risk factors** | | |
| --- | --- | --- |
|  | **OR (95% Cl)** | ***p* Value** |
| **Patients with Hypertension (n=142)** |  |  |
| Age, y | 1.02(0.98 to 1.05) | 0.414 |
| Sex, female | 1.79(0.78 to 4.08) | 0.17 |
| Smoking history | 0.66(0.29 to 1.50) | 0.318 |
| Diabetes | 0.75(0.32 to 1.79) | 0.517 |
| High Fisher grade (3-4) | 2.36(1.00 to 5.58) | **0.05** |
| Total Fazekas score | 1.48(1.13 to 1.93) | **0.004** |
| High CSO-EPVS (n≥10) | 3.26(1.41 to 7.55) | **0.006** |
| High BG-EPVS (n≥10) | 5.26(2.20 to 12.57) | **<0.001** |
| **Patients with Diabetes (n=59)** |  |  |
| Age, y | 1.03(0.95 to 1.11) | 0.484 |
| Sex, female | 1.13(0.30 to 4.31) | 0.86 |
| Smoke | 0.34(0.079 to 1.45) | 0.144 |
| Hypertension | 0.69(0.16 to 2.88) | 0.606 |
| High Fisher grade (3-4) | 0.96(0.22 to 4.10) | 0.95 |
| Total Fazekas score | 4.42(1.52 to 12.85) | **0.006** |
| High CSO-EPVS (n≥10) | 47.78(8.337 to 272.74) | **<0.001** |
| High BG-EPVS (n≥10) | 15.00(4.49 to 64.41) | **<0.001** |
| **Patients with Smoking History (n=96)** |  |  |
| Age, y | 1.00(0.96 to 1.03) | 0.901 |
| Sex, female | 765224925.600 | 0.999 |
| Hypertension | 1.54(0.63 to 3.77) | 0.349 |
| Diabetes | 0.55(0.14 to 2.12) | 0.383 |
| High Fisher grade (3-4) | 1.55(0.60 to 3.97) | 0.364 |
| Total Fazekas score | 1.23(0.93 to 1.62) | 0.151 |
| High CSO-EPVS (n≥10) | 1.78(0.73 to 4.37) | 0.208 |
| High BG-EPVS (n≥10) | 2.44(0.97 to 6.14) | 0.057 |

Abbreviations: aSAH = aneurysmal subarachnoid hemorrhage; BG = basal ganglia; Cl = confidence interval; CSO = centrum semiovale; EPVS = enlarged perivascular spaces; OR = odds ratio.
